# Harmful and potentially harmful constituents (HPHCs) in two novel nicotine pouch products in comparison with regular smokeless tobacco products and pharmaceutical nicotine replacement therapy products (NRTs)

**DOI:** 10.1186/s13065-023-00918-1

**Published:** 2023-03-03

**Authors:** Susanne Back, Anna E. Masser, Lars E. Rutqvist, Johan Lindholm

**Affiliations:** Regulatory & Scientific Affairs, Swedish Match AB, Box 17037, S-104 62 Stockholm, Sweden

**Keywords:** Tobacco harm reduction, Oral tobacco, Swedish snus, Nicotine pouch, Nicotine replacement therapy (NRT), Moist snuff, Chemical analysis, Harmful and potentially harmful constituents (HPHC), GOTHIATEK^®^, Oral tobacco derived nicotine (OTDN) pouches

## Abstract

**Background:**

Tobacco-free nicotine pouches is a novel category of oral nicotine-delivery products. Among current tobacco users such pouches may serve as a low-risk alternative to cigarettes or conventional, tobacco-based oral products e.g., snus and moist snuff. In the United States (U.S.), the market leading nicotine-pouch brand is ZYN®. However, no data on the chemical characteristics of ZYN have been published.

**Methods:**

We screened for 43 compounds potentially present in tobacco products in seven oral nicotine-delivery products: ZYN (dry and moist), snus (General^®^), moist snuff (CRP2.1 and Grizzly Pouches Wintergreen), and two pharmaceutical, nicotine replacement therapy products (NRTs, Nicorette^®^ lozenge and Nicotinell^®^ gum). Thirty-six of the tested compounds are classified as harmful and potentially harmful constituents (HPHCs) by the Center for Tobacco Products at the U.S. Food and Drug Administration (FDA-CTP). Five additional compounds were included to cover the GOTHIATEK^®^ product standard for Swedish snus and the last two compounds were chosen to include the four primary tobacco specific nitrosamines (TSNAs).

**Results:**

The tested products contained nicotine at varying levels. The two ZYN products contained no nitrosamines or polycyclic aromatic hydrocarbons (PAHs) but low levels of ammonia, chromium, formaldehyde, and nickel. In the NRT products we quantified low levels of acetaldehyde, ammonia, cadmium, chromium, lead, nickel, uranium-235, and uranium-238. The largest number (27) and generally the highest levels of HPHCs were quantified in the moist snuff products. For example, they contained six out of seven tested PAHs, and seven out of ten nitrosamines (including NNN and NNK). A total of 19 compounds, none of which were PAHs, were quantified at low levels in the snus product. NNN and NNK levels were five to 12-fold lower in snus compared to the moist snuff products.

**Conclusions:**

No nitrosamines or PAHs were quantified in the ZYN and NRT products. Overall, the number of quantified HPHCs were similar between ZYN and NRT products and found at low levels.

## Background

Long-term epidemiological studies have convincingly shown that use of traditional, tobacco-based Swedish snus is associated with substantially fewer and/or less severe adverse health effects than cigarette smoking [1]. In Sweden, snus has since the early 1970s to a large extent replaced cigarettes, particularly among male tobacco users and is now the dominating tobacco product on the Swedish market [2, 3]. The extensive use of snus instead of cigarettes has contributed to internationally record low rates of smoking and smoking-and tobacco-related disease, a phenomenon often referred to as the “Swedish Experience” in the literature. According to the World Health Organization (WHO) Swedish males have the European Union’s lowest rate of “tobacco-related” mortality [4].

In 2019, eight snus products marketed in the United States (U.S.) were granted a modified risk tobacco product (MRTP) order by the U.S. Food and Drug Administration’s Center for Tobacco Products (FDA-CTP) [5]. The scientific basis for the MRTP order came in part from the “Swedish Experience” [6]. Furthermore, the snus products are manufactured according to a stringent product standard (GOTHIATEK^®^) which includes maximum levels for several constituents classified as harmful and potentially harmful (HPHC) by the FDA [2].

From its launch in 2000, GOTHIATEK covered tobacco specific nitrosamines (TSNAs) (most notably N-nitrosonornicotine (NNN) and 4-(methylnitrosamino)-1-(3-pyridyl)-1-butanone (NNK)), and polycyclic aromatic hydrocarbons (PAHs) (including benzo[a]pyrene (B(a)P)). From a long-term health point of view, NNN, NNK, and B(a)P have historically been regarded as the most problematic HPHCs in snus. Although the GOTHIATEK maximum levels have been gradually lowered over the years, and despite improved manufacturing methods snus products still contain measurable levels of NNN and NNK. However, discontinued use of fire-cured tobacco has led to over 95% lower levels of B(a)P in snus [2, 7]. Moreover, the levels of NNN, NNK and B(a)P are substantially lower in snus than in moist snuff products [8, 9].

In recent years several pouched, nicotine delivery products intended for oral use that do not contain tobacco (in the following referred to as “nicotine pouches”) have become commercially available in Europe and the U.S. A nicotine pouch is used in the same way as snus: it is placed under the upper lip where it delivers nicotine systemically via the oral mucosa. After use, the pouch is discarded. In the U.S., the market leader in this novel category is a product sold under the brand name ZYN® which in 2019 had a market share of 86% [10]. As nicotine pouches do not contain tobacco and the added nicotine has a purity that meets pharmaceutical standards they should, at least in theory, not expose users to the HPHCs that are typically present in tobacco, such as TSNAs.

This paper presents the results of a screening for 43 selected compounds in two nicotine pouch products, ZYN dry and ZYN moist. For comparative purposes, the screening was also performed in one pouched Swedish snus product, two types of moist snuff (loose and pouched), and two pharmaceutical nicotine replacement therapy products (NRTs, lozenge and gum).

## Material and methods

### Tested products

Table 1 summarizes selected characteristics of the tested products.

**Table 1:** Product characteristics

Analyte	Units	ZYN (dry)	ZYN (moist)	NRT (lozenge)	NRT (gum)	Snus (pouch)	Moist snuff (loose)	Moist snuff (pouch)
Product mass	g	0.4	0.8	0.6	1.2	1.0	–	1.3
pH		8.3	8.3	8.6	9.9	8.9	7.7	7.9
Moisture	%	3	37	–	–	51	53	52
Size	mm	14 × 28	13.5 × 34	–	–	18 × 33	–	18 × 44

#### ZYN nicotine pouches

Two variants of ZYN were tested: ZYN dry which has a 3% moisture content and comes in a rectangular pouch made of a non-woven material. The pouch measures 14 × 28 mm and weighs 0.4 g. The pouch contains fillers (maltitol and microcrystalline cellulose), a stabilizer (hydroxypropyl cellulose), pH adjusters (sodium carbonate and sodium bicarbonate), a nicotine salt, food grade flavorings, and a sweetener (acesulfame K).

ZYN moist which has a 37% moisture content and comes in a rectangular pouch made of a non-woven material. The pouch measures 13.5 × 34 mm and weighs 0.8 g. The pouch ingredients are slightly different from that of ZYN dry: water, fillers (microcrystalline cellulose and plant fibers), a humectant (glycerine), pH adjusters (sodium carbonate and calcium chloride), sodium chloride, food grade flavorings, a nicotine solution, a monoglyceride, and a sweetener (acesulfame K).

#### NRTs

Two NRT products were tested, Nicorette Peppermint 2 mg lozenge, and Nicotinell Licorice 2 mg gum. The products weigh 0.6 g and 1.2 g per unit of use, respectively. The lozenge contains nicotine in the form of resinate, fillers (mannitol, xanthan gum, gum arabic, magnesium stearate, hypromellose, titanium dioxide, microcrystalline cellulose, potassium silicate, polysorbate 80), pH adjuster (sodium carbonate), sweetener (sucralose, acesulfame K) and flavorings. The gum contains nicotine polacrilex, chewing gum base, sweetener (acesulfame K, saccharin, sodium saccharin, sorbitol, xylitol, mannitol), pH adjusters (calcium carbonate, sodium carbonate, sodium bicarbonate), flavoring, glycerol, gelatine, titanium dioxide, canauba wax and talcum powder.

#### Swedish snus

The tested snus product was General^®^ Portion Original Large which is one of the eight products for which FDA-CTP issued a MRTP order in 2019. General has a moisture content of 51% and comes in a rectangular pouch made of non-woven material. The pouch measures 18 × 33 mm and weighs 1.0 g. The pouch contains ground, air-cured tobacco, water, sodium chloride, sodium carbonate, humidifying agents, and food-grade flavorings. During manufacturing, the mixture of ground tobacco, water and salt is heat treated (pasteurized) to reduce microbial activity.

#### Moist snuff

Two variants of moist snuff were tested: the Cooperation Centre for Scientific Research Relative to Tobacco (CORESTA) Smokeless Tobacco Reference Product (CRP2.1) [11] which is a non-pouched product with a 53% moisture content. It contains both air-cured and dark fire-cured tobaccos, water, sodium chloride, burley stem, and sodium carbonate. We also tested one of the market-leading pouched products in the U.S., Grizzly Pouches Wintergreen. It has a moisture content of 52%, comes in a 18 × 44 mm pouch made of a non-woven material, and weighs 1.3 g.

The manufacturing processes for both moist snuff products include fermentation of the tobacco.

### Selected compounds

A total of 43 compounds were selected for analysis, 36 of which are classified as HPHCs by the FDA [12]. This included the nine compounds on the FDA-CTP’s list of HPHCs relevant for smokeless tobacco products: acetaldehyde, arsenic, B(a)P, cadmium, crotonaldehyde, formaldehyde, nicotine (total and unprotonated), NNK, and NNN [13]. We also screened for some other compounds covered by the GOTHIATEK standard (aflatoxin B2, G1 and G2, nitrite, and ochratoxin A) [7]. In addition, N-nitrosoanatabine (NAT), and N-nitrosoanabasine (NAB) were included to cover all four, primary TSNAs. All reported results are based on wet weight.

GOTHIATEK also includes maximum levels for a large number of agrochemicals. Analyses of such compounds were considered beyond the scope of the current study.

### Sample handling and analysis

The snus product and the two ZYN products were obtained from Swedish Match Distribution Center, Stockholm, Sweden. The NRT products were obtained from on-line pharmacies in Sweden. CRP2.1 was obtained from North Carolina State University Tobacco Analytical Services Laboratory, North Carolina, U.S. The pouched moist snuff product was purchased from Hardec’s Wholesale, Kentucky, U.S. One batch of each product was analyzed. All analyses were performed within 3 weeks of obtaining the product. Pending analysis, the NRTs and ZYN dry were stored at room temperature whereas ZYN moist, snus and the moist snuff products were kept refrigerated or frozen. Where applicable, we followed the CORESTA Guide No. 11 Technical Guide for Sample Handling of Smokeless Tobacco and Smokeless Tobacco Products [14].

Table 2 lists the tested compounds and provides brief descriptions of the analytical methods. The analyses were performed in triplicate on the entire product including the pouch material, where applicable. The data are presented as the mean and standard deviation of the triplicate (Tables 3–6). Where one or two out of the three replicates have no measurable levels i.e., below limit of quantification (LoQ), the values for the individual replicates were set to 50% of the LoQ for the mean and standard-deviation calculations. Most of the included compounds were analyzed at the external contract laboratory Eurofins, Lidköping, Sweden. Polonium-210 was analyzed at Labstat, Kitchener, Canada. A few compounds that could not be analyzed at Eurofins were analyzed in-house at the Swedish Match Laboratory, Stockholm, Sweden. All methods used by Eurofins and Swedish Match are validated and accredited to ISO 17025 for tobacco products and nicotine pouches. For NRT-matrices, the analytical methods are validated and fit for its intended purpose, but not yet accredited according to ISO 17025. All three laboratories are accredited according to ISO 17025.

**Table 2:** Analytes, method code, and description of analytical methods

Analyte	Method code	Description
Arsenic Cadmium Chromium Lead Nickel Selenium Beryllium	Eurofins: EN ISO 17294–2:2016/EN 13805:2014	Digestion was performed in a microwave oven with a mix of nitric acid, hydrochloric acid, and hydrogen peroxide, followed by detection and quantification on an inductively coupled plasma mass spectrometry (ICP-MS)
Mercury	Eurofins: EN 16277:2012	Digestion was performed according to Annex D of EN16277:2012 with a mix of nitric acid, hydrochloric acid and hydrogen peroxide, followed by detection and quantification using cold vapor atomic fluorescence spectroscopy
Uranium-235 Uranium-238	Swedish Match: FDA method: “CFSAN/ORS/DBC/CHCB April 25, 2011” (ICP-MS technique) (Modified)	The metals were released from a matrix through microwave digestion using Milli-Q water, nitric acid with a concentration of 67–69%, and hydrogen peroxide. The obtained solution was analyzed using an ICP-MS
Polonium-210	Labstat/Maxxam	Detected using alpha emission spectrometry
NAB (N-Nitrosoanabasine) NAT (N-Nitrosoanabatine) NNK (4-(Methylnitrosamino)-1-(3-pyridyl)-1-butanone) NNN (N-Nitrosonornicotine)	Eurofins: In-house LW0A0	Extracted with ethyl acetate in presence of d-labelled specific internal standards, followed by detection and quantification with high-performance liquid chromatography using a C18, 3 µm column and a tandem mass-spectrometry (HPLC–MS/MS), positive polarity
NDMA (N-Nitrosodimethylamine)	Eurofins: In-house LP061	Extracted with ethyl acetate in the presence of a specific internal standard, followed by detection and quantification with HPLC–MS/MS, positive polarity. An HSS T3, 1.8 µm APCI column was used
NDELA (N-Nitrosodiethanolamine)	Swedish Match: In-house, based on [30, 31]	Extracted with Milli-Q water with the addition of an internal standard (NDELA-d8). The water extract was cleaned up using two different SPE-columns following separation and quantification using ultra performance liquid chromatography-MS/MS (UPLC-MS/MS)
NMOR (N-Nitrosomorpholine) NPIP (N-Nitrosopiperidine) NPYR (N-Nitrosopyrrolidine) NSAR (N-Nitrososarcosine)	Swedish Match: In-house, based on [32]	After addition of the internal standards (NSAR-d3, NMOR-d4, NPYR-d4, and NPIP-d4) the nitrosamines were extracted using 2% formic acid in acetonitrile. The extracts were diluted with 2% formic acid in water and filtered. Separation and quantification were performed using UPLC-MS/MS
Benz[a]antracene Benzo[b,k]fluoranthene Chrysene Dibenz[a,h]anthracene Indeno[1,2,3-cd]pyrene Naphthalene	Eurofins: In-house SLF92	Extracted with acetone in presence of specific d-labelled internal standards. The extracts were transferred to a hexane solution, followed by detection and quantification with gas chromatography-mass spectrometry, GC–MS. A J&W DB-5 ms GC column 0.18 µm was used
B(a)P (Benzo[a]pyrene)	Eurofins: In-house LW0R7	Extraction was performed with methanol in presence of a d-labelled specific internal standard, followed by detection and quantification with HPLC-FLD
Nitrite	Eurofins: In-house LW 091	Extracted in Milli-Q water derivatized with sulfanilamide and naphtylethylendiamine hydrochloride and analyzed as a red complex at 540 nm
Acetaldehyde Crotonaldehyde Formaldehyde	Eurofins: CORESTA recommended method No. 86 [33]	Extraction and derivatization were performed in the presence of specific internal standards in a two-phase-system consisting of an aqueous buffer and isohexane using DNPH as a derivatization agent, followed by detection and quantification on UPLC-MS/MS
Aflatoxin B1 Aflatoxin B2 Aflatoxin G1 Aflatoxin G2	Eurofins: EN 14123 (mod)	Extracted using acetonitrile/methanol/water and transferred to a phosphatic buffer saline and cleaned using a monoclonal antibody affinity column. After elution from the column the aflatoxins were post-derivatized followed by detection and quantification using high performance liquid chromatography with fluorescence detection (HPLC-FLD)
Ochratoxin A	Eurofins: NMKL 143	Extracted with a mix of acetonitrile and water, followed by a concentration step on a preparative column based on monoclonal antibody technology. The eluate was subsequently analyzed by liquid chromatography with fluorescence detection
Coumarin	Eurofins: In-house method	Extracted in 50% ethanol in presence of a specific internal standard, followed by detection and quantification on UPLC-MS/MS, positive polarity. A BEH, 1.7 µm column was used
Ethyl carbamate	Eurofins: In-house method	Extracted with Milli-Q water in presence of a specific internal standard, followed by detection and quantification by UPLC-MS/MS, positive polarity. A HSS T3, 1.8 µm column was used
Ammonia	Eurofins: In-house LW0A3	Extracted with Milli-Q water and mixed with salicylate and dichlor-isocyanate in presence of sodium nitroprusside to form a blue complex detectable at 660 nm
Nicotine	Eurofins: Health Canada Official method T301 (Modified)	Extracted with alkaline methanol in presence of a specific d-labelled internal standard
Anabasine Nornicotine	Swedish Match: CORESTA recommended method No. 62 [34] (Modified)	Mixed with sodium hydroxide and extracted using an extraction solution with methyl tert-butyl ether and quinoline as an internal standard. Separation and quantitation were performed using a gas chromatograph fitted with a capillary column and flame ionization detector
pH	Eurofins: CORESTA recommended method No. 69 [35]	Diluted with milli-q water 1:10, stirred for 5 min and measured with pH-meter
Unprotonated nicotine	Swedish Match	Calculated according to [36]

**Table 3:** Analytical results for nitrosamines presented as average ± standard deviation for triplicate measurements

Analyte	Units	Limit of quanification	ZYN (dry)	ZYN (moist)	NRT (lozenge)	NRT (gum)	Snus (pouch)	Moist snuff (loose)	Moist snuff (pouch)
NAB (N-Nitrosoanabasine)	µg/g	0.01	*	*	*	*	0.02 ± 0	0.30 ± 0.02	0.24 ± 0.01
NAT (N-Nitrosoanabatine)	µg/g	0.01	*	*	*	*	0.31 ± 0.02	3.50 ± 0.10	2.43 ± 0.06
NDELA (N-Nitrosodiethanolamine)	ng/g	25	*	*	*	*	*	*	*
NDMA (N-Nitrosodimethylamine)	ng/g	0.20	*	*	*	*	0.24 ± 0.01	7.90 ± 0	2.67 ± 0.06
NMOR (N-Nitrosomorpholine)	ng/g	10	*	*	*	*	*	*	*
NNK (4-(Methylnitrosamino)-1-(3-pyridyl)-1-butanone)	µg/g	0.01	*	*	*	*	0.19 ± 0.01	2.27 ± 0.05	0.92 ± 0.01
NNN (N-Nitrosonornicotine)	µg/g	0.01	*	*	*	*	0.44 ± 0.01	3.63 ± 0.06	2.53 ± 0.06
NPIP (N-Nitrosopiperidine)	ng/g	10	*	*	*	*	*	*	*
NPYR (N-Nitrosopyrrolidine)	ng/g	10	*	*	*	*	*	54 ± 3	*
NSAR (N-Nitrososarcosine)	ng/g	25	*	*	*	*	*	150 ± 17	177 ± 59

^*^ The measured analyte was below the quantification limit

Using CORESTA Recommended Method No. 69 we measured pH to permit estimates of the amount of unprotonated nicotine.

## Results

In total, we analyzed 43 compounds (including nicotine). Table 3 shows the analytical results for nitrosamines, Table 4 for PAHs, Table 5 for heavy metals and radionuclides, and Table 6 for the remaining compounds.

**Table 4:** Analytical results for PAHs presented as average ± standard deviation for triplicate measurements

Analyte	Units	Limit of Quanification	ZYN (dry)	ZYN (moist)	NRT (lozenge)	NRT (gum)	Snus (pouch)	Moist snuff (loose)	Moist snuff (pouch)
Benz[a]antracene	ng/g	30	*	*	*	*	*	553 ± 15	393 ± 6
Benzo[a]pyrene	ng/g	1	*	*	*	*	*	113 ± 6	74 ± 3
Benzo[b,k]fluoranthene	ng/g	30	*	*	*	*	*	270 ± 0	173 ± 6
Chrysene	ng/g	30	*	*	*	*	*	537 ± 21	390 ± 20
Dibenz[a,h]anthracene	ng/g	30	*	*	*	*	*	*	*
Indeno[1,2,3-cd]pyrene	ng/g	30	*	*	*	*	*	41 ± 1	*
Naphthalene	ng/g	30	*	*	*	*	*	70 ± 1	34 ± 1

^*^ The measured analyte was below the quantification limit

**Table 5:** Analytical results for metals and radionuclides presented as average ± standard deviation for triplicate measurements

Analyte	Units	Limit of quanification	ZYN (dry)	ZYN (moist)	NRT (lozenge)	NRT (gum)	Snus (pouch)	Moist snuff (loose)	Moist snuff (pouch)
Arsenic	µg/g	0.050	*	*	*	*	0.062 ± 0.002	0.077 ± 0.004	0.083 ± 0.012
Beryllium	µg/g	0.050	*	*	*	*	*	*	*
Cadmium	µg/g	0.010	*	*	*	0.043 ± 0.002	0.270 ± 0	0.730 ± 0.010	0.670 ± 0.017
Chromium	µg/g	0.050	0.160 ± 0.036	0.099 ± 0.053	*	0.850 ± 0.030	0.523 ± 0.032	0.490 ± 0.060	0.463 ± 0.045
Lead	µg/g	0.020	*	*	*	0.067 ± 0.003	0.177 ± 0.006	0.203 ± 0.012	0.157 ± 0.012
Mercury	µg/g	0.020	*	*	*	*	*	*	*
Nickel	µg/g	0.050	0.067 ± 0.006	*	0.086 ± 0.030	0.243 ± 0.015	0.817 ± 0.046	0.707 ± 0.042	0.800 ± 0.017
Polonium-210	Bq/kg	5.0	*	*	*	*	5.4^a^	9.0^a^	8.1^a^
Selenium	µg/g	0.050	*	*	*	*	0.088 ± 0.006	0.113 ± 0.006	0.067 ± 0.001
Uranium-235	Bq/kg	0.02	*	*	*	0.14 ± 0.04	*	*	*
Uranium-238	Bq/kg	0.25	*	*	*	2.76 ± 0.66	*	*	*

^a^ Based on a single measurement

^*^ The measured analyte was below the quantification limit

**Table 6:** Analytical results for miscellaneous compounds presented as average ± standard deviation for triplicate measurements

Analyte	Units	Limit of Quanification	ZYN (dry)	ZYN (moist)	NRT (lozenge)	NRT (gum)	Snus (pouch)	Moist snuff (loose)	Moist snuff (pouch)
Acetaldehyde	µg/g	1.0	*	*	*	4.7 ± 0.3	10.0 ± 0	5.7 ± 0.2	4.8 ± 0.2
Aflatoxin B1	ng/g	0.1/1.0^a^	*	*	*	*	*	*	*
Aflatoxin B2	ng/g	0.1/1.0^a^	*	*	*	*	*	*	*
Aflatoxin G1	ng/g	0.1/1.0^a^	*	*	*	*	*	*	*
Aflatoxin G2	ng/g	0.1/1.0^a^	*	*	*	*	*	*	*
Ammonia	µg/g	1.0	62 ± 1	66 ± 1	*	4.5 ± 0	847 ± 45	2300 ± 0	3900 ± 100
Anabasine	µg/g	20.0	*	*	*	*	28.5 ± 0.3	54.6 ± 1.5	42.5 ± 0.1
Coumarin	µg/g	0.05	*	*	*	*	*	0.86 ± 0.02	0.65 ± 0.02
Crotonaldehyde	µg/g	0.050	*	*	*	*	*	*	*
Ethyl carbamate	ng/g	30	*	*	*	*	*	*	*
Formaldehyde	µg/g	1.0	10.3 ± 0.6	1.5 ± 0.1	*	*	1.6 ± 0.1	4.3 ± 0.1	2.0 ± 0.1
Nicotine (total)	mg/g	0.01	7.63 ± 0.21	12.00 ± 0	3.20 ± 0.10	1.30 ± 0.10	8.13 ± 0.38	9.97 ± 0.06	8.47 ± 0.12
Nicotine (unprotonated)^b^	mg/g	0.01	4.87 ± 0.36	7.87 ± 0	2.57 ± 0.14	1.28 ± 0.10	7.11 ± 0.28	3.23 ± 0.02	3.65 ± 0.05
Nitrite	µg/g	1.0	*	*	*	*	*	*	*
Nornicotine	µg/g	50	*	*	*	*	162 ± 2	220 ± 4	129 ± 0
Ochratoxin A	ng/g	0.50	*	*	*	*	0.86 ± 0.05	*	2.90 ± 0.26

^a^ Aflatoxin quantification limits were 0.1 ng/g for the NRT lozenge and 1.0 ng/g for the other tested products

^b^ Unprotonated nicotine was calculated based on pH for the different products (ZYN dry 8.3, ZYN moist 8.3, NRT (lozenge) 8.6, NRT (gum) 9.9, snus 8.9, moist snuff (loose) 7.7, moist snuff (pouch) 7.9)

^*^ The measured analyte was below the quantification limit

As expected, total and unprotonated “free” nicotine at varying levels was quantified in all tested products (Table 6).

In the two types of ZYN nicotine pouches, 38 of the 43 analyzed compounds were below the respective level of quantification. Most notably, this included all tested nitrosamines and PAHs. In addition to nicotine, a total of three HPHCs were found in both the ZYN dry and ZYN moist products: formaldehyde (10.3 µg/g and 1.5 µg/g, respectively), chromium (0.160 µg/g and 0.099 µg/g, respectively), and ammonia (62 µg/g and 66 µg/g, respectively). Traces of nickel, just above the quantification limit, were found in ZYN dry (0.067 µg/g).

In addition to nicotine, nickel at a low level (0.086 µg/g) was the only compound found in the NRT lozenge product. In the NRT gum product eight compounds in addition to nicotine were quantified. This included cadmium (0.043 µg/g), chromium (0.850 µg/g), lead (0.067 µg/g), nickel (0.243 µg/g), acetaldehyde (4.7 µg/g), ammonia (4.5 µg/g), and the uranium isotopes ^235^U (0.14 Bq/kg) and ^238^U (2.76 Bq/kg). Notably, no nitrosamines or PAHs were found in either NRT product.

All compounds quantified in the ZYN products were also found in snus and the moist snuff products, but the levels found were vastly different. In general, the ZYN and NRT products contained the lowest levels followed by snus and moist snuff. Compared to snus, 69% and 81% less chromium were found in ZYN dry and ZYN moist, respectively. ZYN dry contained 92% less nickel than snus. For ammonia, 93% and 92% less were found in ZYN dry and ZYN moist, respectively. ZYN moist and snus contained comparable levels of formaldehyde whereas the level in ZYN dry were about 5 times higher than in snus.

Where lead, cadmium, nickel, and ammonia were quantified in the NRT products, their levels were 63–99% lower compared to snus. In contrast, 63% more chromium were found in the NRT gum than snus. The NRT gum contained half as much of acetaldehyde compared to snus. Also, the two uranium isotopes were only found in the NRT gum.

The snus product contained 19 of the 43 compounds. In addition to nicotine, this included five nitrosamines (NAB, NAT, NDMA, NNK, and NNN), six heavy metals (arsenic, cadmium, chromium, lead, nickel, selenium), acetaldehyde, ammonia, anabasine, formaldehyde, nornicotine, ochratoxin A, and a polonium isotope (^210^Po).

A total of 27 and 26 compounds were quantified in the loose and pouched moist snuff products, respectively. Apart from nicotine, this included six nitrosamines (NAB, NAT, NDMA, NNN, NNK, and N-nitrososarcosine (NSAR)) in both products and N-Nitrosopyrrolidine (NPYR) in the loose moist snuff. The levels of NNN and NNK were about five to 12-fold higher than in the snus product. A total of six and five PAHs were quantified in the loose and pouched moist snuff products, respectively. The quantified heavy metals were the same, and at comparable levels as those found in snus: arsenic, cadmium, chromium, lead, nickel, and selenium. Other compounds present in the moist snuff products were acetaldehyde, ammonia, anabasine, coumarin, formaldehyde, nornicotine, and ^210^Po. In addition, the pouched moist snuff product contained ochratoxin A.

## Discussion

Tobacco harm reduction is defined as “decreasing total morbidity and mortality, without completely eliminating tobacco and nicotine use” [15]. In practice, a prerequisite for tobacco harm reduction is therefore availability of nicotine products which entail lower risks than cigarettes. The MRTP order for Swedish snus issued by the FDA-CTP in 2019 authorized the following statement to be used for marketing purposes: “Using General snus instead of cigarettes puts you at a lower risk of mouth cancer, heart disease, lung cancer, stroke, emphysema, and chronic bronchitis”. This list of “tobacco-related” adverse health outcomes thus includes the conditions that contribute the most to the excess morbidity and mortality documented among cigarette smokers [16]. However, some compounds are naturally present in tobacco, formed during curing, or snus production. This includes nitrosamines that have been classified as potentially carcinogenic. From that perspective a tobacco-free nicotine pouch should, at least in theory, represent an improvement in terms of exposure to nitrosamines and other tobacco-related compounds.

This paper reports the results from a screening of 43 compounds in two types of ZYN products, as one of the crucial initial steps in a full risk assessment. For comparative purposes the same screening was performed in some other oral, nicotine delivery products: Swedish snus, moist snuff, an NRT lozenge and an NRT gum.

The fewest compounds (nicotine included) were quantified in the NRT lozenge (two), followed by the ZYN moist and ZYN dry products (four and five, respectively), and the NRT gum (nine). Notably, we found no measurable levels of nitrosamines or PAHs in any of these products.

In the snus product we quantified 19 compounds, including NNN and NNK but no PAHs. In contrast, the two loose and pouched moist snuff products contained 27 and 26 compounds, respectively. These included several PAHs, presumably originating from the dark fire-cured tobacco. The NNN and NNK levels in the moist snuff products were five- to 12-fold higher than in the snus product. The substantial differences between snus and moist snuff may be explained by differences in the selection of raw tobaccos (fire-cured tobacco is not used in snus production) as well as different manufacturing processes: snus is heat treated so that the finished product has a very low microbial activity, whereas moist snuff is fermented [2, 14]. Fermentation may allow for bacterial production of nitrosamines.

Few HPHCs were found and at low levels in ZYN dry and ZYN moist suggesting that the products have a low toxicity. Consistent with our findings in vitro toxicology studies showed that nicotine pouches are associated with less cytotoxicity and has less impact on biological processes compared to conventional smokeless tobacco products [17–19]. Furthermore, extracts from nicotine pouches were not mutagenic nor genotoxic [19]. The fact that use of conventional, smokeless tobacco products typically entails simultaneous exposure to a larger number and broader variety of HPHCs than is the case with nicotine pouches may help to explain these findings.

We found considerable variations in total as well as unprotonated, “free nicotine” among the tested products. “Free nicotine” has been suggested to more accurately than total nicotine reflect the potential for nicotine uptake with oral, nicotine delivery products [20]. However, it may not be an ideal proxy for the total uptake among consumers as the concept of “free nicotine” does not consider the amount of nicotine that is extracted from the product. Reliable assessments of the nicotine pharmacokinetics of individual products therefore require clinical studies that directly measure the uptake of nicotine.

Clinical data on the nicotine pharmacology of ZYN dry showed a similar nicotine-delivery profile and comparable nicotine exposure to that with snus and moist snuff [21]. In contrast, nicotine pouches were associated with less positive subjective effects and slower nicotine delivery when compared to combustible cigarettes. This suggests that nicotine pouches might have a lower abuse liability [22, 23].

A similar study reported by Azzopardi and colleagues [24] analyzed 26 compounds in a range of nicotine delivery products including a nicotine pouch (LYFT^®^), Swedish-type snus, moist snuff, and NRTs (lozenge and gum). Our results confirm this study as no TSNAs, or B(a)P could be quantified in ZYN or NRT products and extend findings to include more HPHCs from the compound classes nitrosamines, PAHs, metals, and radionuclides.

The data presented here is limited to the HPHC content of nicotine pouches in relation to other smokeless nicotine/tobacco products. Although such data may suggest the potential for adverse health effects among individual consumers, a full risk assessment requires more data. Recent in vitro studies [17–19] and clinical studies [21, 25–29] have provided important insights. However, long-term data on consumer perceptions and behaviors and most importantly epidemiological data are needed for a full risk assessment.

## Conclusions

A screening for 43 HPHCs in two variants of the nicotine pouch product ZYN showed that only few HPHCs were quantified and all at consistently low levels. These findings were similar to those for the tested NRT products. Notably, nitrosamines or PAHs were not found in either the ZYN or NRT products.

## Data Availability

All datasets used are available upon reasonable request to the corresponding author. For more details regarding analysis methods please contact the respective lab.

## Abbreviations

B(a)P: Benzo[a]pyrene
CORESTA: Cooperation Centre for Scientific Research Relative to Tobacco
CRP2.1: CORESTA Smokeless Tobacco Reference Product
FDA-CTP: The Center for Tobacco Products at the United States’ Food and Drug Administration
GC–MS: Gas chromatography-mass spectrometry
HPHCs: Harmful and potentially harmful constituents
HPLC-FLD: High performance liquid chromatography with fluorescence detection
HPLC–MS/MS: High-performance liquid chromatography with tandem mass-spectrometry
ICP-MS: Inductively coupled plasma-mass-spectrometry
LoQ: Limit of Quantification
MRTP: Modified risk tobacco product
NAB: N-Nitrosoanabasine
NAT: N-Nitrosoanatabine
NDMA: N-Nitrosodimethylamine
NNK: 4-(Methylnitrosamino)-1-(3-pyridyl)-1-butanone
NNN: N-Nitrosonornicotine
NRTs: Nicotine replacement therapy products
NSAR: N-nitrososarcosine
NPYR: N-nitrosopyrrolidine
PAHs: Polycyclic aromatic hydrocarbons
^210^Po: Polonium-210
TSNA: Tobacco-specific nitrosamines
^235^U: Uranium-235
^238^U: Uranium-238
U.S: United States
UPLC-MS/MS: Ultra-performance liquid chromatography with tandem mass spectrometry
WHO: The World Health Organization
